# Chitosan and Curcumin Nanoformulations against Potential Cardiac Risks Associated with Hydroxyapatite Nanoparticles in Wistar Male Rats

**DOI:** 10.1155/2021/3394348

**Published:** 2021-07-29

**Authors:** Israa F. Mosa, Haitham H. Abd, Abdelsalam Abuzreda, Amenh B. Yousif, Nadhom Assaf

**Affiliations:** ^1^Department of Nanotoxicology and Animal Physiology, Institute of Graduate Studies and Research, Alexandria University, Alexandria, Egypt; ^2^Department of Animal Physiology, Ministry of Education, Anbar, Iraq; ^3^University of Benghazi, Department of Health Safety and Environment (HSE), Arabian Gulf Oil Company (AGOCO), Benghazi, Libya; ^4^Department of Family and Community Medicine, Faculty of Medicine, University of Benghazi, Benghazi, Libya; ^5^Department of Toxicology, Ministry of Education, Anbar, Iraq

## Abstract

Nanoparticle-induced cardiovascular diseases have attracted much attention. Upon entering the blood circulation system, these particles have the potency to induce cardiomyocytes, leading to cardiac failure or myocardial ischemia, and the molecular mechanism remains to be completely clarified. In this study, the cardiac toxicity of rats orally exposed to hydroxyapatite nanoparticles (HAPNPs) has been observed through an increase in myocardial infarction serum markers including CK-MB and alterations in routine blood factors, expression of apoptosis-related protein P53, and increased levels of serum inflammatory markers represented by the tumor necrosis factor alpha and Interleukin-6, as well as a decline in heart antioxidant enzymes and reduced glutathione level, while an induction in lipid peroxidation and nitric oxide has been observed, as well as notable histological and histochemical alterations in the heart of these animals. mRNA and protein expressions of vascular endothelial growth factor (VEGF-A), cyclooxygenase-2 (COX-2), and atrial natriuretic factor (ANF) were elevated in the myocardium. However, the coadministration of chitosan nanoparticles (CsNPs) and/or curcumin nanoparticles (CurNPs) successfully modulated these alterations and induced activation in antioxidant parameters. The present data suggest that HAPNPs-induced apoptosis via the mitochondrial pathway may play a crucial role in cardiac tissue damage and the early treatment with CsNPs and CurNPs may protect the heart from infarction induced by HAPNPs toxic effect.

## 1. Introduction

Hydroxyapatite (HAP) is a biocompatible material that presents as the mineral or inorganic phase in hard tissues (bones and teeth) of vertebrates. Therefore, it is aligned with the improvement of novel preparation methods where it can be biomedically applied in order to repair hard tissues or utilized as a drug delivery vehicle [1]. These include antibiotics, enzymes, anticancer drugs, growth factors, and antigens delivery for slow vaccination release. In recent years, hydroxyapatite nanoparticles (HAPNPs), whose diameters are less than 100 nm have been reported to have better biocompatibility and bioactivity than their bulk counterpart [2]. Hence, HAPNPs' safety and toxicity concerns are growing regardless of their favorable potency in several biomedical applications.

Due to HAPNPs nanoscale particle size, they have entered substantially all fields of life, which has raised concerns regarding their toxicity. They can internalize the cells and interact with biological molecules, therefore, affect the cells in a detrimental manner through altering cell response, leading to toxicological response [3]. With the increasing applications of these nanoparticles, especially in diagnostic purposes and cardiovascular medicine [4], hence entering the body through other routes of exposure can indeterminately target the cardiovascular system [5]. Besides direct cardiac exposure to them, the cardiovascular system is also affected by NPs secondary effects on other organs, leading to systemic cardiovascular toxicity and atherosclerosis, as well as increased risk of cardiopulmonary toxicity [6]. Furthermore, reactive oxygen species (ROS) generated during prolonged oxidative stress are accountable for numerous cardiovascular diseases such as heart failure, cardiomyopathy, myocardial infarction, atherosclerosis, and cardiac hypertrophy. Cellular “redox homeostasis” mostly preserves the healthy physiology in endothelial cells and cardiac myocytes [7].

Hence, preventive strategies have been suggested against cardiac toxicity. As reported by the current suggested cardiotoxicity mechanism, the compound that gives free radical scavenging ability is considered as a good contestant to be rated as a supportive treatment for heart injury throughout oxidative damage. Further, during redundant oxidative stress, body's endogenous system fails to retain normal physiology. For this reason, antioxidant co-supplementation is required, as they are capable of scavenging free radicals and other toxic radicals. Numerous antioxidants such as chitosan, beta carotene, curcumin, lycopene, resveratrol, quercetin, and vitamin E have shown therapeutic and preventive impacts in various cardiovascular diseases forms [8].

However, the rapid systemic clearance and poor solubility of curcumin limit its use in clinical practices. Therefore, curcumin nanoformulations have overcome this problem and improved their physicochemical properties relevant to their performance [9]. Curcumin nanoparticles (CurNPs) have gained immense attention for their better bioavailability as nanotechnology has improved curcumin water solubility, bioavailability, and absorbability. For this reason, it can effortlessly penetrate in and pass through organisms' cell membranes and promptly interact with various biological systems [10].

Moreover, many authors used chitosan as a natural antioxidant product against nanoparticles prospected toxicity as several studies have shown that nanoform of chitosan (CsNPs) has remarkable biological activities such as antioxidant and anti-inflammatory, antitumoral, and antibacterial activities and also enhances immune function. Additionally, CsNPs and CurNPs showed synergic antioxidant ability when administered alone or together in reversing HAPNPs induced nephron and gastrointestinal toxicity at several levels [11, 12].

Nanoparticles usage has much to offer, particularly in the field of cardiovascular medicine. Hence, the present study aimed to outline HAPNPs adverse effects and their associated cardiac toxicity through evaluating heart biochemical parameters as it has been suggested that oxidative stress and inflammation are the crucial factors accountable for adverse effects induced by ultrafine particles, also, analyzing the histological and immunohistochemical architecture and eventually inspecting the role of CsNPs and CurNPs in inverting the prospected toxicity.

## 2. Materials and Methods

### 2.1. Characterization of Hydroxyapatite, Chitosan, and Curcumin Nanoparticles

Nanoparticles used in the present study underwent transmission electron microscope (TEM) for morphology examination.

#### 2.1.1. Transmission Electron Microscope Analysis

HAPNPs, CsNPs, and CurNPs were examined for morphology using the transmission electron microscope, TEM (JEOL JEM 2100Plus operated at 80 kV, Japan), where samples were placed on carbon-coated copper grids and left 5 min to dry at room temperature; the extra solution was distanced by blotting paper. The JEOL JEM 2100Plus is a versatile TEM for both large-scale 2D screening as well as tomography. It runs Serial EM and thus is capable of automated multiposition acquisitions. Depending on the type and thickness of the specimen, the acceleration voltage can be chosen from 80 to 200 kV.

### 2.2. Tested Compounds and Doses

Hydroxyapatite nanoparticles (HAPNPs) were purchased from Nanoshel LLC, USA; chitosan nanoparticles (CurNPs) and chitosan nanoparticles (CsNPs) with Mw 310–375 kDa and degree of deacetylation (DD 75%) were purchased from Nanotech Egypt. The dosage of HAPNPs (300 mg/kg bw) was chosen according to the Mosa method [11], while the doses of CsNPs (280 mg/kg bw) and CurNPs (15 mg/kg bw) have followed the dosing criteria in the following studies [13, 14], respectively.

### 2.3. Ethics Statement

Laboratory animals and sample collection were performed following the protocols and barrier system conducted in compliance with Alexandria University's national ethical standards and endorsed by the Institutional Animal Care and Use Committee (IACUC) to reduce animals' suffering.

### 2.4. Animal Grouping and Dose Administration

Eighty Wistar male rats weighing 170–175 g were obtained from the animal house colony of the faculty of Medicine, Alexandria University, Egypt. Rats were housed in cages with *ad libitum* access to a certified rodent basal diet and sterilized tap water maintained at controlled temperature (20–25°C) and 40–70% relative humidity in a room free of any kind of chemical contaminants. Taken into consideration that water, food consumption, and body weights (gain or loss) were recorded weekly till the end of the experiment. All animals had no previous abnormal clinical conditions before the study. After two weeks of lab acclimatization, rats were randomly divided into eight equal groups with ten rats each. The first group was served as control. Rats in the second group were administered CsNPs (280 mg/kg bw) by gavage whilst animals in the third group received CurNPs (15 mg/kg bw); the fourth group received CsNPs and CurNPs; the fifth group received HAPNPs (300 mg/kg bw); the sixth group received CsNPs and HAPNPs; the seventh group received CurNPs and HAPNPs; the eighth group was served as the combination as it has received CsNPs, CurNPs, and HAPNPs. Animals were orally treated with the respective doses daily for 45 days.

### 2.5. Preparation of Samples

At the end of the experiment, rats of each group were sacrificed under ether anesthesia and blood samples were collected in heparin collecting tubes for serum separation by centrifuging at 860 ×*g* for 20 minutes, while plasma was kept at -80°C for biochemical analyses. Cardiac samples were collected, weighed, and washed with chilled saline solution (0.9%), and then connective tissues and adhering fats were immediately removed. The first section was used for DNA fragmentation assay, whereas the second part was used for gene expression analysis. Additionally, the third part was immersed instantly in formalin in order to attain histological and immunohistochemical analysis, and the last part was minced and homogenized to yield (10%, w/v) homogenates.

### 2.6. Measured Parameters

#### 2.6.1. Assay of Analysis of COX-2, VEGF, and ANF Gene Expression Using RT-PCR

Total RNA was extracted from cardiac tissues with the RNeasy Extraction Kit (Qiagen®, USA). Quantitative RT-PCR assays were performed using Rotor-Gene SYBR Green RT-PCR Kit (Qiagen®, USA) on Rotor-Gene Q (Qiagen®, Valencia, CA, USA). Quantitative RT-PCR amplification conditions started with initial reverse transcription for the synthesis of cDNA for 10 minutes at 55°C and the resultant cDNA was then amplified by 40 cycles of PCR as follows: Denaturation at 95°C for 5 seconds, annealing at 55°C for 15 seconds and extension at 60°C for 15 seconds. GAPDH, the housekeeping gene, was used as a reference gene for normalization. Primers used for rat genes are presented in Table 1. The values of the threshold cycle (Ct) were determined by Rotor-Gene Q-Pure Detection version 2.1.0 (build 9) (Qiagen®, USA). For each gene, the relative change in mRNA in samples was determined using the 2^−ΔΔCt^ method (4) and normalized to the housekeeping gene GAPDH.

#### 2.6.2. Index of Lipid Peroxidation and Nitric Oxide Assay

The lipid peroxidation process results in malondialdehyde (MDA) end products, whereas its quantification is generally considered as lipid peroxidation activity marker. MDA levels were determined in the heart using thiobarbituric acid-reactive substances (TBARS) as in Tappel and Zalkin's proposed method, whereas nitric oxide (NO) level was assayed by Montgomery and Dymock.

#### 2.6.3. Assay of Antioxidant Determination

Reduced glutathione (GSH) content was determined and the method utilized metaphosphoric acid for protein precipitation and 5,5′-dithiobis (2-nitrobenzoic acid) (DTNB) for color development and its density was measured at 412 nm. GSH was assayed following the Jollow method. Total antioxidant capacity (TAC) in heart homogenates was assayed in accordance with Koracevic. The activity of superoxide dismutase (SOD) was measured as stated by Misra and Fridovich method and the assay procedure involves the inhibition of epinephrine autooxidation in an alkaline medium (pH 10.2) to adrenochrome, which is markedly inhibited by the presence of SOD. Epinephrine was added to the assay mixture containing tissue supernatant and the change in extinction coefficient was followed at 480 nm in a spectrophotometer. Glutathione peroxidase (GPx) activity was determined following the Chiu method. Glutathione S-transferase (GST) activity was analyzed based on the Habig method. GST catalyzes the conjugation reaction with glutathione in the first step of the mercapturic acid synthesis. The activity of GST was measured in heart homogenate and *p*-nitrobenzyl chloride was used as substrate. The absorbance was measured spectrophotometrically at 310 nm using UV-double beam spectrophotometer. Catalase (CAT) activity was assayed as per the Luck method. The CAT activity was measured spectrophotometrically at 240 nm by calculating the rate of degradation of H_2_O_2_, the substrate of the enzyme. The above-mentioned assays were assessed in consistency with Biodiagnostic Kit, Egypt manual instruction.

#### 2.6.4. Determination of CK-MB and LDH Activities

Serum was obtained after rats' lives were laboratory ceased. Levels of serum myocardial enzymes, including creatine kinase-muscle/brain (CK-MB), were assayed using an ELISA kit (cat.no. K777; BioVision, Inc.), and lactate dehydrogenase (LDH) was measured using a commercial kit (cat. no. 283001; Spectrum Diagnostic Company, Eg.) based on the method of Bais and Philcox.

#### 2.6.5. Lipid Profile Assessment

Stored plasma samples were examined for total lipids (cat. no. 8.05.36.0.0250; Atlas Medical UK), cholesterol (cat. no. 11805), triglycerides (TAG; cat. no. 11828), and high-density lipoprotein cholesterol (HDL-c; cat. no. 11557) using (BioSystems S.A.) commercial kits. In addition, very low-density lipoprotein-cholesterol (vLDL-c) was calculated by driving the values of TAG by a factor of five. Low-density lipoprotein-cholesterol (LDL-c) was determined by the following formula: LDL-c = cholesterol-(vLDL-c + HDL-c).

#### 2.6.6. Tumor Suppressor Gene p53 and Cytokine Assessment

Tumor suppressor gene p53 was quantified using ELISA kits (Active Motif co. 1914, Palomar Oaks Way, Suite 150, Carlsbad, CA 92008 USA), while rat tumor necrosis factor-alpha (TNF-*α*) was tested by ELISA kits for TNF-*α* quantitative measurement (Abcam co., UK). Also, Interleukin-6 (IL-6) was assayed using Enzyme-linked Immunosorbent Assay (ELISA) kit for IL-6 *in vitro* quantitative measurement in heart tissue homogenates (Kamiya Biomedical C., 12779 Gateway Drive, Seattle, WA98168).

### 2.7. Histopathological Analysis of the Heart

Tissues taken from rats'hearts were cut and instantly fixed in 10% formalin for 14–18 h and then dehydrated through ascending grades of xylene, and alcohol was used for dehydration until they reached the absolute alcohol (1 hour). Tissues were embedded in xylene and molten wax (1 : 1) for 10 minutes, then inserted in paraffin wax 56°C, and then sectioned to obtain 4–6 *μ*m thickness by Rotary microtome. The paraffin blocks were mounted in a microtome where successive sections adhere to form a straight ribbon. Subsequently, the outcome was stained with Haematoxylin and Eosin to investigate histopathological changes using a light microscope with 400x magnification as described in the Drury method [15].

### 2.8. Proliferating Cell Nuclear Antigen Immunoreactivity Measurement

Heart distribution of PCNA receptor subunits was examined in deparaffinized sections with the desired tissue thickness for immunohistochemistry (IHC), which is 5 *μ*m using an Avidin-Biotin-Peroxidase (IHC) method (Elite-ABC; Vector Laboratories, CA, USA); anti-PCNA monoclonal antibody (dilution 1 : 100; DAKO Japan Co, Tokyo, Japan) was employed. Briefly, sections were deparaffinized, rehydrated, washed in phosphate buffered saline (PBS) (3 × 5 min), and peroxidase activity was quenched using 0.3% H_2_O_2_ in methanol for 30 min. Subsequently, samples were washed in PBS and incubated with blocking solution at room temperature for 10 min. After rinsing with PBS, sections were incubated with biotinylated mouse anti-PCNA primary antibody in a moist chamber for 30–60 min and then rinsed with PBS. Samples were incubated with Streptavidin Peroxidase at room temperature for 10 min and washed with PBS. The antibody-peroxidase complex was developed using DAB chromogen at 18–24°C for 2–5 min. Finally, the sections were washed with PBS, counterstained with Haematoxylin and Eosin for 1 min, washed with tap water, then PBS for 30 seconds, dehydrated through ascending grades of alcohol, dilapidated in xylene, and cover-slipped with Mount-Quick (Daido Sangyo, Tokyo).

### 2.9. Statistical Analysis and Statistics

Data were reported as means ± standard error (SE). Statistical analysis for all studied parameters was performed using the general linear model (GLM) produced by SAS Institute, Inc. Duncan's New Multiple Range Test was used to test the significance of the differences between the individual treatments following Duncan's new multiple range test. Values were considered significantly different when *p* < 0.05.

## 3. Results

**Figure 1:**
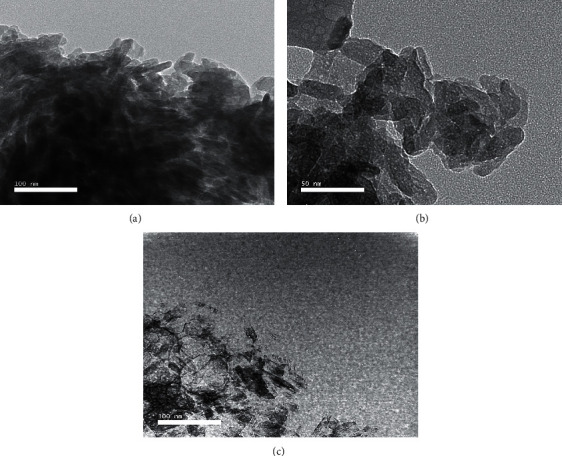
TEM micrographs of needle like nano-HAP.

**Figure 2:**
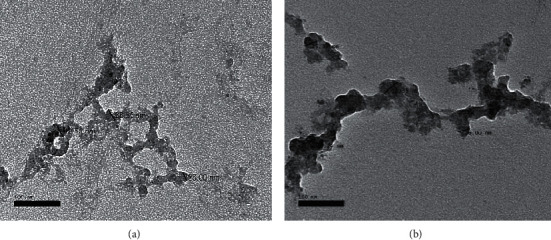
TEM micrographs and size distribution of CsNPs.

**Figure 3:**
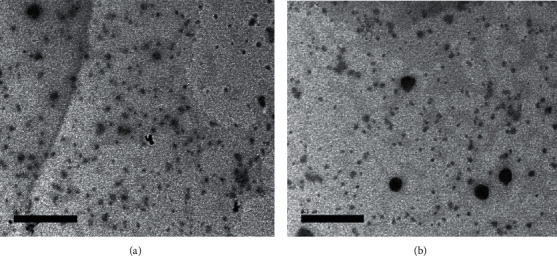
TEM micrographs and size distribution of CurNPs.

The structure and morphology of hydroxyapatite, chitosan, and curcumin-nanoparticles were further confirmed by transmission electron microscopic (TEM) micrographs as shown in Figures 1–3 as it has confirmed the presence of needle-like shape crystals of HAPNPs at different scale bars 50–100 nm with an average particle size of 10.48 nm (Figure 1).  Figure 2 has provided evidence of spherical and smooth surface morphology of CsNPs with an average particle size of 35.63 nm. While in Figure 3, CurNPs were presented in a well-defined crystalline structure with an average particle size of 0.04 *μ*m.

**Figure 4:**
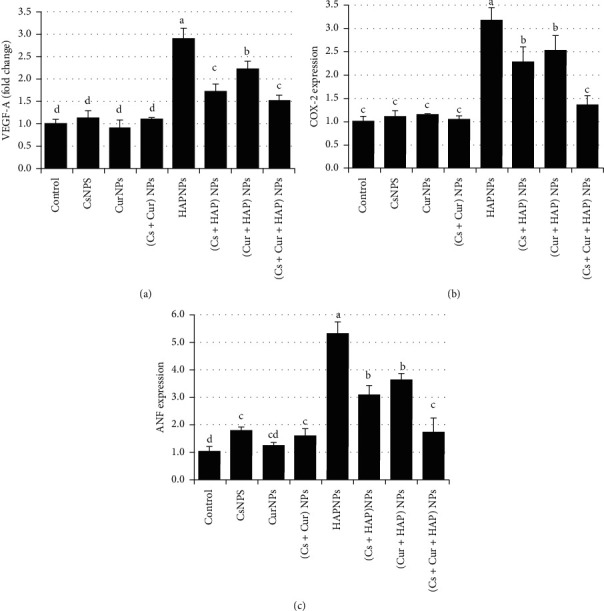
Mean values ± SE of heart levels of vascular endothelial growth factor-A (VEGF-A), cyclooxygenase-2 (COX-2 expression), and atrial natriuretic factor (ANF expression).

Furthermore, VEGF-A, COX-2, and ANF mRNA and proteins expressions are outlined in Figure 4 and Table 2. HAPNPs-treated group showed significant induction in VEGF-A, COX-2, and ANF, while rats that received CsNPs and/or CurNPs showed nonsignificant high expression of VEGF-A, COX-2, and ANF. On the other hand, the presence of CsNPs and CurNPs along with HAPNPs in the combination group has inhibited the induction in VEGF-A, COX-2, and ANF expression and utterly normalized their values as shown in Figure 4.

**Figure 5:**
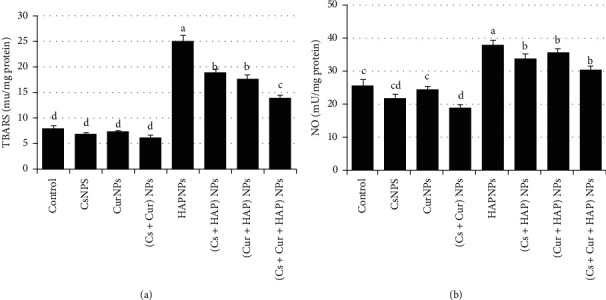
Mean values ± SE of heart thiobarbituric acid-reactive substances and nitric oxide.

In addition, treatment with HAPNPs demonstrated a significant (*p* < 0.05) elevation in TBARS and NO levels in heart tissue compared to any other group. At the same time, the presence of CsNPs and/or CurNPs in the combination group has significantly corrected and declined the elevation in TBARS and NO content (Figure 5 and Table 3).

**Figure 6:**
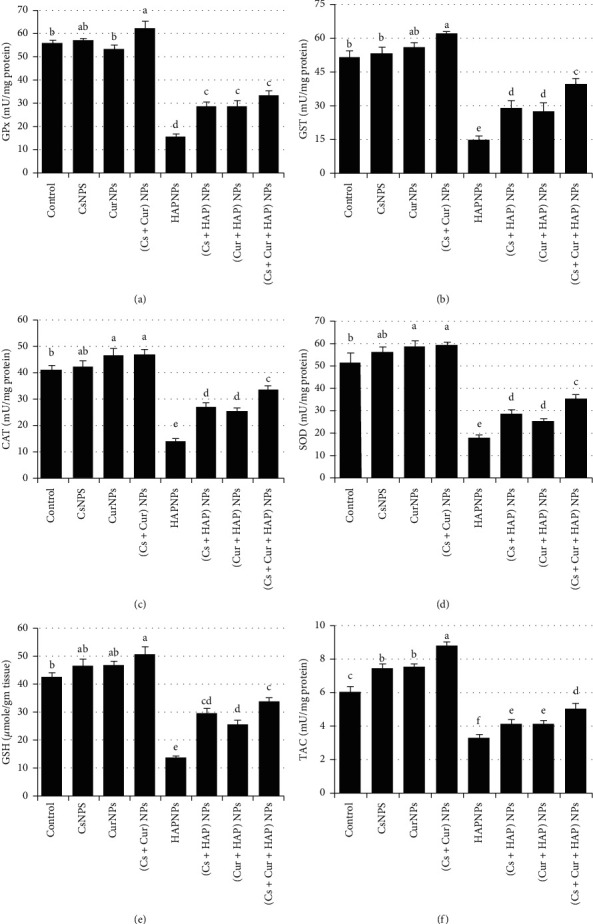
Mean values ± SE of heart glutathione peroxidase, glutathione S-transferase, catalase, superoxide dismutase, total antioxidant capacity, and reduced glutathione.

As shown in Figure 6, the activities of heart antioxidant parameters (GPX, GST, CAT, SOD, GSH, and TAC) have shown a significant (*p* < 0.05) decline in HAPNPs-treated group. Of note, the treatment with CsNPs and/or CurNPs was capable of regulating their values (Table 4).

**Figure 7:**
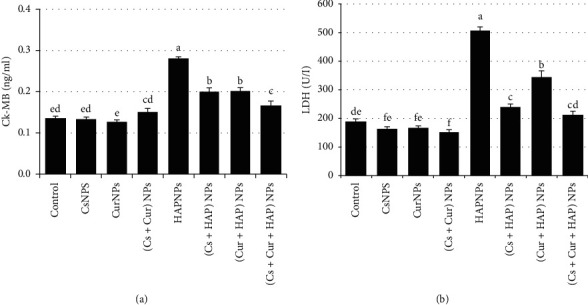
Mean values ± SE of heart creatine kinase-muscle/brain and lactate dehydrogenase.

**Figure 8:**
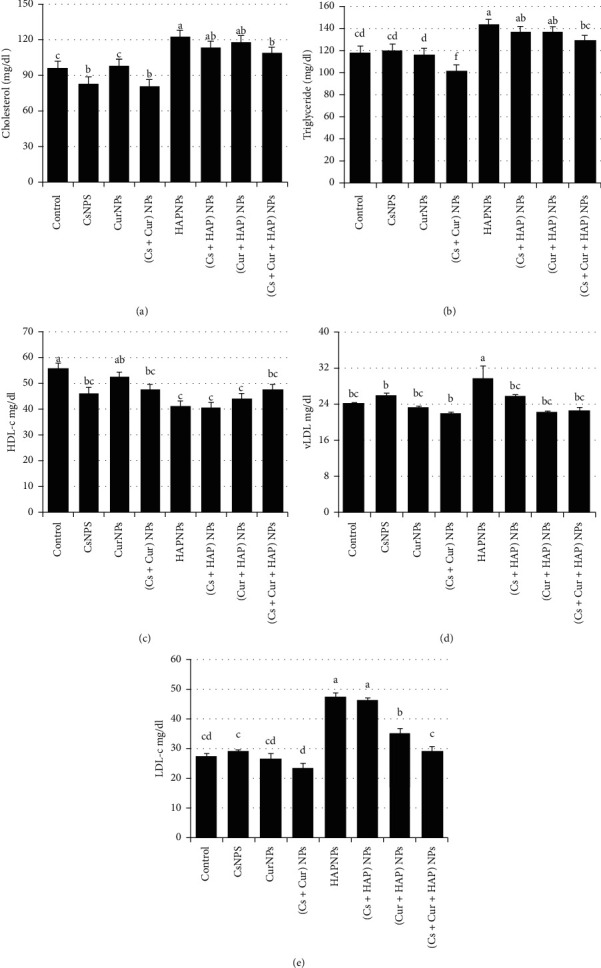
Mean values ± SE of cholesterol, triglyceride, high-density lipoprotein cholesterol, very low-density lipoprotein cholesterol, and low-density lipoprotein cholesterol.

Foremost, CK-MB is considered an important cardiac biomarker used to assist myocardial infarction diagnoses. CK-MB has shown an elevation in rats' plasma and cardiac tissues treated with HAPNPs (Figure 7), while the presence of CsNPs and/or CurNPs has minimized this elevation (Table 5). Further, the plasma levels of total lipid, triglycerides, cholesterol, LDL-c, vLDL-c, and LDH of rats exposed to HAPNPs were significantly higher compared to control, whilst significantly lower levels of HDL-c as represented in Figure 8 and Table 6.

**Figure 9:**
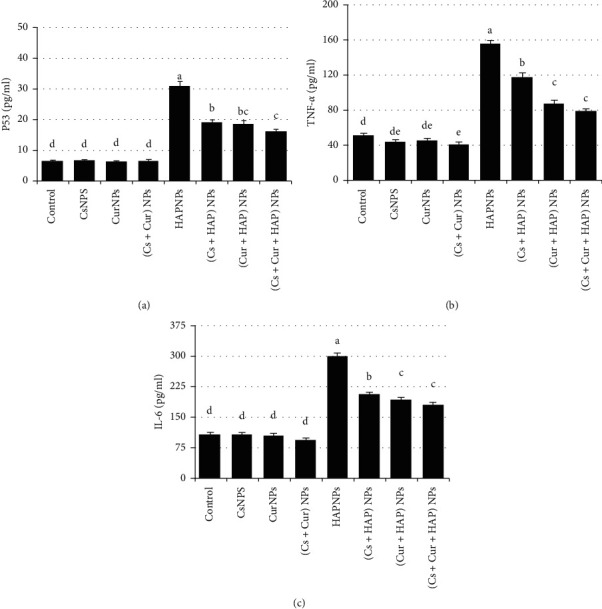
Mean values ± SE of heart levels of tumor suppressor P53, tumor necrosis factor-*α,* and interleukin-6.

Tumor suppressor p53 controls the cell cycle to suppress tumors production, also acts as an apoptosis inductor and genome guard. Tumor necrosis factor-*α* (TNF-*α*) and interleukin-6 (IL-6) play an important role in oxidative stress and tissue injury. Results suggested that the group treated with HAPNPs revealed significantly high levels of p53, TNF-*α,* and IL-6 in cardiac tissues. While CsNPs and CurNPs have completely reversed the increase in their levels as shown in Figure 9 and confirmed in Table 7.

**Figure 10:**
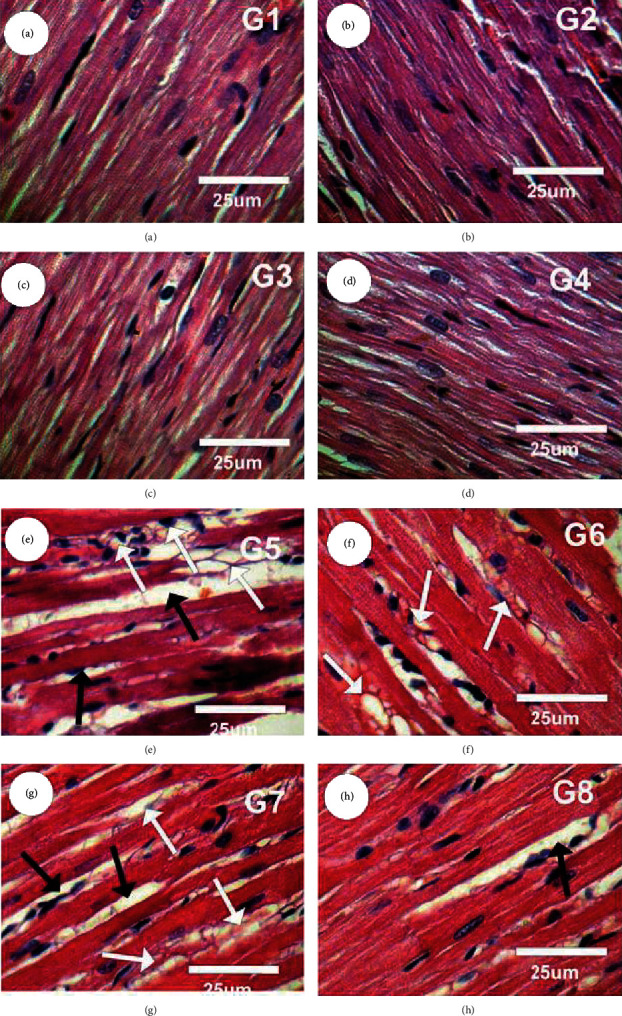
(a–h) Photomicrographs of rat left ventricle section in different experimental groups stained with Haematoxylin and Eosin.

Figures 10(a)–10(d) represent histological alterations of rats' heart stained with H&E in the following groups, starting with control (G1), CsNPs-treated group (G2), CurNPs-treated group (G3), and both CsNPs and CurNPs-treated group (G4), revealing a normal myofibrillar structure with striations. Moreover, heart sections of rats treated with HAPNPs (G5) revealed marked hydrophobic changes of myofibrillar structure with striations, strong myocardial hypertrophy, marked cytoplasmic vacuoles, and moderate degeneration (Figure 10(e)). On the other hand, rat left ventricle section in HAPNPs, and CsNPs-treated group (G6) revealed mild to moderate hydrophobic changes of myofibrillar structure with striations, mild myocardial hypertrophy, and cytoplasmic vacuoles as represented in Figure 10(f). Left ventricle section in HAPNPs- and CurNPs-treated group (G7) revealed moderate hydrophobic changes of myofibrillar structure with striations, moderate myocardial hypertrophy, and mild cytoplasmic vacuoles as in Figure 10(g). In contrast, left ventricle section in the combination group treated with HAPNPs, CsNPs, and CurNPs (G8) revealed mild tissue injury with a few myocardial atrophy and a few cytoplasmic vacuoles as in Figure 10(h).

**Figure 11:**
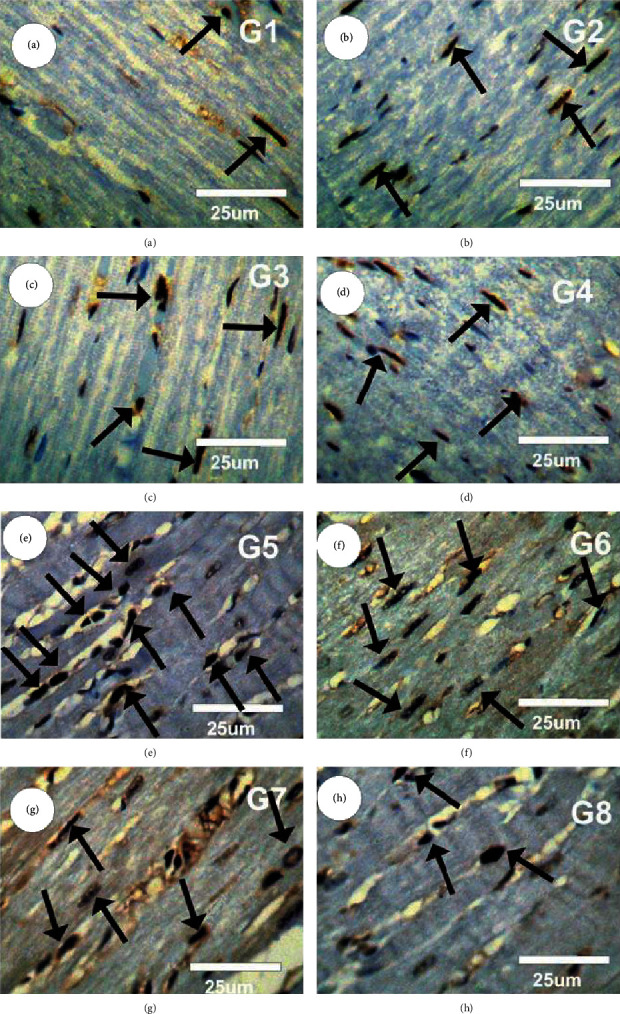
(a–h) Photomicrographs of rat myocardium section in different experimental groups stained with PCNA-ir.

Figures 11(a)–11(h) show PCNA immunoreactivity (PCNA-ir) distributions in cardiac muscle tissues in different experimental groups. Myocardium in the control group (G1); CsNPs (G2), CurNPs (G3), and both CsNPs- and CurNPs-treated group (G4) revealed mild positive reaction for PCNA (grade 1) as documented in Figures 11(a)–11(d), while severe positive reactions for PCNA-ir (grade 4) were spotted in the myocardium section in HAPNPs-treated group (G5) as in Figure 11(e). Moreover, mild to moderate positive reactions for PCNA-ir (grade 3) were detected in the myocardium of the group treated with both HAPNPs and CsNPs (Figure 11(f)). Also, moderate positive reactions for PCNA-ir (grade 3) were spotted in the myocardium of HAPNPs- and CurNPs-treated group (Figure 11(g)). Mild positive reactions for PCNA-ir (grade 2) were observed in the myocardium of the combination group which received HAPNPs, CsNPs, and CurNPs (Figure 11(h)).

## 4. Discussion

With respect to toxicology, aiming to the small size of nanoparticles, it is possible to provoke variety of interactions of cell-material that would induce toxicological impact. They can also travel through the cell barrier and allocate systemically from the injection site. Systemic intake of nanoparticles via lymph can arise following ingestion. In addition, they can be distributed entirely across the organism through the blood circulation, and afterward can be taken up by the heart, kidney, liver, spleen, and other vital organs [16]. Genotoxicity, inflammation, cell injury, inhibition of oxidative stress, and cell division through the formation of ROS are notably elucidated to be significant causes concerning nanoparticles safety prospects [17].

Hydroxyapatite nanoparticles have demonstrated favorable medical findings, whereas a number of reports have indicated that HAPNPs can produce debris resulting in inflammation which is acknowledged as a foreign substance by the immune system that later starts inflammatory cells recruitment such as macrophages, blood monocytes, and neutrophils [18, 19]. Hence, the internalization of HAPNPs is linked to cardiac-toxicity, since these particles have shown multiple cytotoxic impacts, which have prompted a rising concern about their prospected safety. Numerous toxicological studies offered sufficient evidence for HAPNPs cytotoxicity, owing to cellular oxidative stress induction [20]. Importantly, Mosa quantitatively declared that orally administrated HAPNPs were distributed everywhere in the body within blood circulation but mainly accumulated in the kidney and stomach, producing apoptosis [11].

When free radicals start attacking their subcellular and cellular components, oxidative DNA damage takes place. The innate self-repair cells mechanisms can outline such damages to some extent. However, DNA bears vast oxidative damage when exposed to excessive free radicals. These damages are commonly considered irretrievable and are widely known to provoke irreversible lesions in DNA function and structure. Eventually, all these facts hinder the transfer of genes and protein expressions, resulting in genotoxicity and impairment to the physiological function of the organism [21].

Oxidative stress increase is accountable for inducing MAPK signaling pathway and increasing key-molecules transcription levels, in particular NF-*κ*B and Nrf2. These factors, in return, could trigger a series of proinflammatory mediators of mRNA expression which are associated with various inflammatory diseases [22]. At the same time, ROS plays a vital role in the modification of the cardiomyocyte microenvironment. Exposure to nanoparticles might result in cardiotoxicity through excessive production of ROS. Moreover, a study in ischemia reperfusion injury demonstrated that altered redox homeostasis and an increase in ROS levels are in charge of myocardial damage [23]. Hence, the accumulation of these oxidative damage forms leads to genotoxicity and DNA mutations which might induce cell apoptosis or malignant mutation.

Accordingly, MDA is the lipid peroxidation marker that is used to evaluate lipid peroxidation due to increased oxidative stress following HAPNPs prospected cardiotoxicity, exhibiting the oxidative stress status. Redundant generation of ROS has been intimately associated with vascular dysfunction, myocardial damage, apoptosis, and necrosis [24]. Recent studies indicated that nanoparticles act as a mediator for ROS generation as one of the mechanisms which lead to cardiotoxicity [25]. NO and TBARS increased levels are considered markers of lipid peroxidation following HAPNPs exposure. Nitric oxide present in cardiac tissues contributes to HAPNPs induced cardiotoxicity since NO released from cardiac endothelial cells and/or generated within cardiac myocytes has an important autocrine/paracrine effect on myocardial function [26]. Additionally, NO may react with superoxide, generating peroxynitrite, which is an effective and aggressive cellular oxidant. The resultant reactive nitrogen species (RNS) or ROS attack the cellular membrane phospholipids provoking lipid peroxidation. This might underlie the elevated levels of MDA, which is one of lipid peroxidation metabolites. On the other hand, Borra reported that CurNPs administration has decreased MDA levels due to its aptitude to reduce hydrogen peroxide induced lipid peroxidation and inhibit the activity of NO synthase by decreasing the production of NO [27].

Moreover, it has also been demonstrated that NPs precipitation in cardiac tissues causes recruitment of inflammatory cells, which in return induce ROS and cytokines generation, either stimulating or harming resident lung cells [28]. TNF-*α* is not expressed in normal cardiomyocytes, but after myocardial infarction, the anoxia and ischemia activate cardiomyocytes and myocardial mononuclear macrophages, which will in its turn generate TNF-*α* in large amounts in the infarction boundary zone in the myocardium. The present study has confirmed the proinflammatory effect of HAPNPs where high levels of TNF-*α* and IL-6 were identified in cardiac tissues. In line with these results, an expectational rise in serum proinflammatory biomarkers, particularly with regard to IL-6, TNF-*α,* and C-reactive protein in rats treated with ZnO NPs, was reported. The induction of these biomarkers might play an important role in ZnO NPs-induced cardiotoxicity. In cultured cardiac myocytes, TNF-*α* induced ROS production results in DNA damage [29]. Also, TNF-*α* up-regulation can lead to an elevation in the level of other cytokines, in particular IL-6, which is generally known as the chief stimulator of CRP production [30]. Moreover, another simulating study implemented by Mosa concluded that HAPNPs subchronic oral exposure caused proinflammatory status indicated by a distinct elevation in IL-6 and TNF-*α* levels, where the elevation in TNF-*α* levels is associated with nourishing and enhancing neutrophils recruitment in inflammation sites [12]. Accordingly, these proinflammatory factors induce DNA damage such as DNA point mutations, chromosomal fragmentation, DNA inhibition, formation, and repair of methylation patterns, which might result in DNA adducts formation and gene expressions alterations [31].

Furthermore, P53 is an exemplary marker for HAPNPs biological safety assessment. Former studies showed that HAPNPs have induced the production of intracellular ROS and activated p53, which could be responsible for DNA damage and cell apoptosis [12]. In addition to this, the recent results have revealed that HAPNPs were accompanied by p53 upregulation that might be triggered by a range of cellular stresses such as oxidative stress and DNA damage, while many reports attested that p53 plays a significant role in apoptosis induction [32].

Moreover, HAPNPs have considerably hindered the main processes of antioxidants in cardiac tissues in particular with regards to antioxidant enzymes SOD, GST, GPx, CAT, TAC, and GSH system. Oxidative damage indices on DNA molecules and lipids suggest that cardiac tissues suffer from oxidative stress damage not only as a consequence of increased ROS generation but also due to the deterioration of the antioxidant mechanism induced by HAPNPs exposure. Consequently, the prooxidant effect of HAPNPs is becoming increasingly obvious. On the other hand, the depletion of GSH in cardiac tissues results in cellular defense deterioration against ROS and might lead to peroxidative injury because GSH acts as a vital aqueous-phase nonenzymatic antioxidant and plays a significant role in cell protection against ROS and oxidative stress, and additionally fundamental antioxidant enzymes cofactor participating in cellular redox reactions [33].

Serum lactate dehydrogenase (LDH) is an enzyme that is classified as a cardiotoxicity marker. After a myocardial injury, CK-MB and LDH activities in the blood indicate the damage level in cardiac muscular tissue. The elevation in serum LDH activity might be connected with the immense free radicals elevation and their influence on the cellular membrane resulting in LDH leakage from cardiomyocytes impaired membranes within the circulation. This risk is advocated by CK-MB's significant increase in plasma and cardiac activity along with cardiac tissue histological malformation, particularly with regard to myocardial degeneration and congestion. CK-MB increased activity in cardiac tissues due to HAPNPs exposure might result in disturbance of CK's distinctive role in the excitation-contraction coupling mechanism. Elevated levels of serum CK-MB and LDH manifest early and late cardiac injury. These results demonstrated that HAPNPs could attenuate myocardial injury. The results of the present study are in line with the findings. Nemmar declared that Fe_2_O_3_NPs resulted in CK-MB levels elevation in plasma and heart tissues [34]. Similarly, Shen stated that Fe_2_O_3_NPs attack myocardium muscles via oxidative stress-mediated iron toxicity, inducing cardiac function impairment and myocardial damage [35]. Furthermore, Du stated that silica nanoparticles increased CK-MB and LDH serum levels and induced cardiotoxicity. Consequently, they were capable of crossing the alveolar-capillary barrier into the circulation [25]. Additionally, in compliance with the present results, Boarescu reported that CurNPs were capable of maintaining cardiomyocytes' normal structure, increasing their vitality and minimizing the elevation in CK-MB and LDH plasma cardiac enzyme markers following myocardial injury [36].

Additionally, besides HAPNPs proinflammatory and prooxidant effects, they also play an important role as an atherogenic factor and might induce specific metabolic modifications. The atheroprotective effects are obtained from inhibition of LDL oxidation, hence reducing cardiovascular disease risk [37]. Hereafter, the blot results demonstrated that exposure to HAPNPs caused depletion of HDL-c level and elevation of cholesterol, triglycerides total lipids, and LDL-c; these are considered powerful atherogenic factors that disposed rats towards the development of cardiovascular diseases.

Angiogenesis is constrained by numerous antiangiogenic and proangiogenic factors. Among various angiogenesis-stimulating factors, vascular endothelial growth factor (VEGF) is one of the most effective and predominant [38]. Within the proangiogenic factors, VEGF-A is the main regulator of vascular permeability that exerts multiple functions, including stimulation of angiogenesis, vasculogenesis, and inflammation [39]. It plays a vital role in cardiac contractility, wound healing, and cardiac morphogenesis across the myocardium also in the differentiation and proliferation of a range of normal and malignant tissues and cells. On the other hand, VEGF-A in high levels are identified in numerous cardiovascular diseases (CVD) and are closely linked with disease severity and poor prognosis. In the rat model, it has been reported that VEGF-A activated the gene expressions involved in myocardial metabolism and contractility and also inhibited cardiomyocytes' apoptosis [40]. By means of PCR and ELISA methods, our data provided experimental evidence that the level of VEGF-A was upregulated in the serum of rats intoxicated with HAPNPs and this could be one of the mechanisms by which HAPNPs restricts aneurysm growth, while the coadministration of CsNPs and/or CurNPs markedly reduced the imposing elevation in these biomarkers. Furthermore, these agents were efficient in mitigating the angiogenic marker to a normal level.

Accordingly, Zhang demonstrated that AuNPs cytotoxicity in subcutaneous tissues on vascular endothelium was intermediated by direct endothelium death as a consequence of the migration of macrophages through the vascular wall or through macrophages deactivation, which produces VEGF-A [41]. However, in a number of instances, the balance between angiogenesis inhibitors and stimulators is amended on behalf of excessive angiogenic signaling, which eventually drives pathological angiogenesis. Pathologic angiogenesis promotes several diseases such as cancer, inflammatory disorders, and ocular neovascularization [42].

Moreover, COX-2 is still claimed as a proinflammatory antagonist and an adequate target for chronic inflammatory diseases treatment. Also, it is expressed clearly in endothelial cells within tumors and plays a vital role in tumor growth regulation and angiogenic and inflammatory processes in tumor tissues. COX-2 angiogenic effects are intermediated mainly by arachidonic metabolism products. Mechanistically, COX-2 or VEGF-A expression is precisely aligned with endothelial cells angiogenesis [43–45]. However, the present results deliver spectacular insights into how HAPNPs can target various aspects of angiogenic signals by vascular endothelial growth through upregulating VEGF-A and COX-2 expressions.

ANF is a 28 amino acid circulating peptide with natriuretic/diuretic and vasorelaxant properties discharged from human and animal atria by stimuli strongly related to atrial size or pressure increase [46]. Atrial peptides are kept in secretory granules, mostly in the atria, and are discharged by a range of stimuli. ANF may serve as an essential regulatory aptitude in cardiovascular function maintenance. Besides the endocrine and renal actions of this hormone, ANF possesses the capability to mitigate systemic arterial pressure. The exogenously administered and endogenously produced role of ANF in heart failure congestive syndrome provides an area of current research. Various animal studies have shown that increases in atrial volume, pressure, and contraction are connected to an increase in stored ANF release. The present details showed that the rise in plasma ANF was concurrent with cardiotoxicity development. Preliminary studies by Ladenson documented that progressive ANF circulating levels increased in rats suffering from hyperthyroidism [47]. Moreover, Mori reported that neonatal rat cardiomyocytes increased ANF discharge after stimulation with thyrotropin releasing hormone [48].

Moreover, Monma documented that the most common histological spots where hydroxyapatite deposit are kidneys, heart, lungs, and stomach [49]. In our blot results, at the histological level, HAPNPs revealed marked hydrophobic changes of myofibrillar structure with striations, strong myocardial hypertrophy and marked cytoplasmic vacuoles, which are in compliance with Yousef who reported that AgNPs induce cellular alteration and inflammation in cardiac tissues and myocardial degeneration which is characterized by loss of cross striation [50]. Additionally, proliferating cell nuclear antigen immunoreactivity (PCNA-ir) was considered to provide another facet into HAPNPs toxicity mechanism. In accordance with the present results, HAPNPs oral administration induced severe positive reactions for PCNA-ir in the myocardium section. These findings evidently demonstrate that simultaneous exposure to HAPNPs resulted in greater ramifications to the heart. Also, PCNA overexpression reaffirms the histopathological alterations in cardiac tissues.

In the present study, curcumin and chitosan nanoforms were utilized as a cardioprotective agent to overcome native particles' poor water solubility. The enhanced water solubility of both nanoparticles improves their pharmacokinetics and bioavailability characteristics. In return, it boosts their therapeutic effectiveness. Here, the daily treatment with CsNPs and CurNPs for 45 days prevented HAPNPs induced oxidative stress through ameliorating the cardiac lipid peroxidation and NO increase, and also GSH elevation approximately to control values. They hinder lipid peroxidation using linoleate, which is capable of forming and oxidizing fatty acid radicals and also serving as NO scavengers through blocking the enzyme that generates it, thus employing a promoter activity [51]. Various studies have indicated CurNPs' antioxidant activity. Khadrawy stated that CurNPs were capable of reducing the oxidative stress induced by the hippocampus and cisplatin in the cortex [52]. Additionally, Benzer reported that the curcumin cardioprotective effect was a consequence of its antiapoptotic, anti-inflammatory, and antioxidant properties [53]. As Menon and Sudheer confirmed the strong anti-inflammatory action of CurNPs through the suppression of inflammatory enzymes such as lipoxygenase 5 and COX-2, which are the key enzymes of arachidonic acid pathways which are involved in human cancer development, also proinflammatory cytokines such as interleukin-1, 2, 6, 8, and 12, tumor necrosis factor *α*, monocyte chemo-attractant protein 1, nuclear factor *κ*B, and transcription factors [54].

CurNPs antioxidant activity is ascribed to its activity to scavenge free radicals [9] and its ability to potentiate GSH synthesis [55]. This, in turn, explains CurNPs ability to hinder the production of free radicals and lipid peroxidation increase. Furthermore, the antioxidant effect of CurNPs and CsNPs could stop HAPNPs from provoking harmful effects on the cardiovascular system. This might prevent LDH leakage from myocytes and its increase in blood; also hinder HAPNPs inhibitory effect on Na, K-ATPase might help in ionic gradients restoration on both sides of the myocardium to its normal level. These results might be ascribed to CurNPs antioxidant capacity, which acts as a suppressing factor leading to the alleviation of ROS damaging effects produced in the heart due to HAPNPs interactions. Moreover, our results showed that CsNPs and CurNPs prevented cytokines elevation since they can reduce the inflammatory responses, a critical pathway in the regulation of transcription of proinflammatory related genes [56–65].

In conclusion, the present study showed that HAPNPs had induced damage in the cardiovascular system via different pathways including oxidative DNA damage, free radicals generation, induction of inflammatory cytokines, and inhibition of antioxidant mechanisms, which may assume an important role in the corresponding cardiac adverse effects. In addition, chitosan and/or curcumin in nanoform mediated protection against HAPNPs-induced cardiotoxicity with strong anti-inflammatory effect in addition reversed the alterations in heart histology and lipid profiles. Hence, these findings might suggest beneficial effects of CsNPs and CurNPs cosupplementation against the inflammatory response following cardiac infarction induced by HAPNPs.

## Figures and Tables

**Table 1:** Reverse-transcription reaction component.

Gene	Primer sequence	
VEGF-A	F:	5′-TGGACCCTGGCTTTACTG-3′
	R:	5′-GGACGGCTTGAAGATATACTC-3′

COX-2	F:	5′-TGCGATGCTCTTCCGAGCTGTGCT -3′
	R:	5′-TCAGGAAGTTCCTTATTTCCTTTC-3′

ANF	F:	5′-CGTATACAGTGCGGTGTCCAAC-3′
	R:	5′-CGAGAGCACCTTCTCTCTGAGA-3′

GAPDH	F:	5′-GGGTGTGAACCACGAGAAATA-3′
	R:	5′-AGTTGTCATGGATGACCTTGG-3′

**Table 2:** Mean values ± SE of heart levels of vascular endothelial growth factor-A (VEGF-A), cyclooxygenase-2 (COX-2 expression), and atrial natriuretic factor (ANF expression).

Experimental groups	Parameter		
	VEGF-A	COX-2	ANF
Control	1.0 ± 0.05^d^	1.00 ± 0.062^c^	1.00 ± 0.102^d^
CsNPs	1.1 ± 0.09^d^	1.11 ± 0.077^c^	1.76 ± 0.083^c^
CurNPs	0.9 ± 0.10^d^	1.14 ± 0.022^c^	1.20 ± 0.074^cd^
(Cs + Cur) NPs	1.1 ± 0.02^d^	1.04 ± 0,047^c^	1.59 ± 0.137^c^
HAPNPs	2.9 ± 0.14^a^	3.15 ± 0.166^a^	5.27 ± 0.273^a^
(Cs + HAP) NPs	1.7 ± 0.10^c^	2.27 ± 0.191^b^	3.08 ± 0.203^b^
(Cur + HAP) NPs	2.2 ± 0.11^b^	2.51 ± 0.195^b^	3.61 ± 0.138^b^
(Cs + Cur + HAP) NPs	1.5 ± 0.08^c^	1.36 ± 0.113^c^	1.69 ± 0.304^c^

Mean values within a column not sharing common superscript letters (a, b, c, d, e, and f) were significantly different, *p* < 0.05. VEGF-A: vascular endothelial growth factor-A; COX-2: cyclooxygenase-2; ANF: atrial natriuretic factor.

**Table 3:** Mean values ± SE of heart thiobarbituric acid-reactive substances and nitric oxide.

Experimental groups	Parameter	
	TBARS (mU/mg protein)	NO (mU/mg protein)
Control	8.1 ± 0.41^d^	25.5 ± 1.63^c^
CsNPs	6.9 ± 0.26^d^	21.8 ± 1.11^cd^
CurNPs	7.4 ± 0.07^d^	24.5 ± 0.79^c^
(Cs + Cur) NPs	6.2 ± 0.41^d^	19.0 ± 0.75^d^
HAPNPs	25.0 ± 1.18^a^	37.8 ± 1.38^a^
(Cs + HAP) NPs	18.9 ± 0.78^b^	33.6 ± 1.38^b^
(Cur + HAP) NPs	17.7 ± 0.66^b^	35.5 ± 1.13^b^
(Cs + Cur + HAP) NPs	13.9 ± 0.49^c^	30.2 ± 1.17^b^

Mean values within a column not sharing common superscript letters (a, b, c, d, and e) were significantly different, *p* < 0.05. TBARS: thiobarbituric acid-reactive substances; NO: nitric oxide.

**Table 4:** Mean values ± SE of heart glutathione peroxidase, glutathione S-transferase, catalase, superoxide dismutase, reduced glutathione, and total antioxidant capacity.

Experimental groups	Parameter					
	GPx (mU/mg protein)	GST (mU/mg protein)	CAT (mU/mg protein)	SOD (mU/mg protein)	GSH (mU/mg protein)	TAC (mU/mg protein)
Control	55.4 ± 1.53^b^	51.3 ± 2.54^b^	41.1 ± 1.53^b^	51.5 ± 4.30^b^	42.1 ± 1.65^b^	6.0 ± 0.35^c^
CsNPs	56.9 ± 1.06^ab^	52.9 ± 2.70^b^	42.2 ± 2.08^ab^	56.1 ± 2.30^ab^	46.1 ± 2.36^ab^	7.4 ± 0.30^b^
CurNPs	53.2 ± 1.67^b^	55.5 ± 2.05^ab^	46.5 ± 2.26^a^	58.6 ± 2.68^a^	46.4 ± 1.45^ab^	7.5 ± 0.19^b^
(Cs + Cur) NPs	62.0 ± 3.26^a^	61.4 ± 1.14^a^	46.8 ± 1.65^a^	59.0 ± 1.66^a^	50.3 ± 2.56^a^	8.7 ± 0.26^a^
HAPNPs	15.6 ± 1.33^d^	14.7 ± 1.63^e^	14.1 ± 0.73^e^	17.8 ± 1.30^e^	13.6 ± 0.51^e^	3.3 ± 0.18^f^
(Cs + HAP) NPs	28.7 ± 1.72^c^	28.8 ± 3.04^d^	26.9 ± 1.25^d^	28.4 ± 1.92^d^	29.4 ± 1.53^cd^	4.1 ± 0.23^e^
(Cur + HAP) NPs	28.6 ± 2.31^c^	27.3 ± 3.54^d^	25.4 ± 1.11^d^	25.2 ± 1.15^d^	25.3 ± 1.52^d^	4.1 ± 0.18^e^
(Cs + Cur + HAP) NPs	33.7 ± 1.48^c^	39.6 ± 2.10^c^	33.6 ± 1.25^c^	35.3 ± 1.80^c^	33.4 ± 1.40^c^	5.0 ± 0.27^d^

Mean values within a column not sharing common superscript letters (a, b, c, d, e, and f) were significantly different, *p* < 0.05. GPx: Glutathione peroxidase; GST: glutathione S-transferase; CAT: catalase; SOD: superoxide dismutase; GSH: reduced glutathione; TAC: total antioxidant capacity.

**Table 5:** Mean values ± SE of serum levels of CK-MB and LDH.

Experimental groups	Parameter	
	CK-MB (ng/ml)	LDH (U/l)
Control	0.13 ± 0.005^ed^	190.0 ± 6.68^de^
CsNPs	0.13 ± 0.004^ed^	163.5 ± 6.33^fe^
CurNPs	0.13 ± 0.002^e^	165.8 ± 5.58^fe^
(Cs + Cur) NPs	0.15 ± 0.006^cd^	152.0 ± 5.47^f^
HAPNPs	0.28 ± 0.006^a^	505.8 ± 12.08^a^
(Cs + HAP) NPs	0.20 ± 0.009^b^	241.5 ± 6.95^c^
(Cur + HAP) NPs	0.20 ± 0.007^b^	346.0 ± 17.90^b^
(Cs + Cur + HAP) NPs	0.17 ± 0.009^c^	211.8 ± 10.21^cd^

Mean values within a column not sharing common superscript letters (a, b, c, d, and e) were significantly different, *p* < 0.05. CK-MB: creatine kinase-muscle/brain, LDH: lactate dehydrogenase.

**Table 6:** Mean values ± SE of cholesterol, triglyceride, HDL-c, vLDL-c, and LDL-c.

Experimental groups	Parameter				
	Cholesterol (mg/dl)	TAG (mg/dl)	HDL-c (mg/dl)	vLDL-c (mg/dl)	LDL-c (mg/dl)
Control	96.0 ± 2.46^c^	118.3 ± 1.73^cd^	55.8 ± 3.59^a^	24.2 ± 0.20^bc^	27.5 ± 0.79^cd^
CsNPs	82.5 ± 2.55^b^	120.3 ± 3.70^cd^	46.3 ± 2.18^bc^	26.1 ± 0.35^b^	29.7 ± 0.45^c^
CurNPs	97.8 ± 3.18^c^	116.5 ± 1.47^d^	52.3 ± 1.44^ab^	23.3 ± 0.35^bc^	26.7 ± 1.52^cd^
(Cs + Cur) NPs	80.5 ± 2.70^b^	101.8 ± 3.22^f^	47.5 ± 2.21^bc^	22.0 ± 0.28^b^	23.7 ± 1.36^d^
HAPNPs	122.0 ± 2.91^a^	143.0 ± 2.72^a^	41.0 ± 1.32^c^	29.6 ± 2.86^a^	47.4 ± 1.42^a^
(Cs + HAP) NPs	122.8 ± 1.92^ab^	136.5 ± 4.99^ab^	40.5 ± 2.09^c^	25.8 ± 0.31^bc^	46.1 ± 0.96^a^
(Cur + HAP) NPs	117.3 ± 3.20^ab^	136.3 ± 4.13^ab^	44.0 ± 0.97^c^	22.3 ± 0.35^bc^	35.1 ± 1.62^b^
(Cs + Cur + HAP) NPs	107.8 ± 4.03^b^	128.5 ± 1.29^bc^	47.5 ± 1.39^bc^	22.7 ± 0.58^bc^	29.2 ± 1.61^c^

Mean values within a column not sharing common superscript letters (a, b, c, d, e, and f) were significantly different, *p* < 0.05. TAG: triglycerides; HDL-c: high-density lipoprotein-cholesterol; vLDL-c: very low-density lipoprotein-cholesterol; LDL-c: low-density lipoprotein-cholesterol.

**Table 7:** Mean values ± SE of heart levels of tumor suppressor P53, tumor necrosis factor-*α*, and interleukin-6.

Experimental groups	Parameter		
	p53 (pg/ml protein)	TNF-*α* (pg/ml tissue)	IL-6 (pg/ml tissue)
Control	6.6 ± 0.33^d^	52 ± 1.32^d^	105 ± 4.3^d^
CsNPs	6.9 ± 0.12^d^	44 ± 2.03^de^	105 ± 5.1^d^
CurNPs	6.5 ± 0.22^d^	45 ± 1.84^de^	103 ± 4.8^d^
(Cs + Cur) NPs	6.8 ± 0.38^d^	41 ± 2.22^e^	93 ± 3.9^d^
HAPNPs	30.8 ± 1.73^a^	155 ± 3.52^a^	300 ± 6.0^a^
(Cs + HAP) NPs	19.2 ± 0.72^b^	117 ± 4.70^b^	207 ± 3.3^b^
(Cur + HAP) NPs	18.5 ± 1.25^ab^	87 ± 3.80^c^	192 ± 6.0^c^
(Cs + Cur + HAP) NPs	16.5 ± 0.82^c^	79 ± 1.67^c^	180 ± 5.6^c^

Mean values within a column not sharing common superscript letters (a, b, c, d, e, and f) were significantly different, *p* < 0.05. P53: tumor suppressor protein; TNF-*α*: tumor necrosis factor-*α*; IL-6: interleukin-6.

## Data Availability

No data were used to support this study.
